# Tick-Borne Encephalitis Virus Antibodies in Roe Deer, the Netherlands

**DOI:** 10.3201/eid2502.181386

**Published:** 2019-02

**Authors:** Jolianne M. Rijks, Margriet G.E. Montizaan, Nine Bakker, Ankje de Vries, Steven Van Gucht, Corien Swaan, Jan van den Broek, Andrea Gröne, Hein Sprong

**Affiliations:** Dutch Wildlife Health Centre, Utrecht, the Netherlands (J.M. Rijks, M.G.E. Montizaan, N. Bakker, A. Gröne);; National Institute for Public Health and the Environment, Bilthoven, the Netherlands (A. de Vries, C. Swaan., H. Sprong);; Sciensano, Brussels, Belgium (S. Van Gucht);; Utrecht University, Utrecht (J. van den Broek, A. Gröne)

**Keywords:** tick-borne encephalitis virus, TBEV, viruses, tick-borne encephalitis, antibodies, public health, surveillance, epidemiologic monitoring, geographic mapping, encephalitis/meningitis, sentinel species, vector-borne infections, roe deer, Capreolus capreolus, zoonoses, the Netherlands

## Abstract

To increase knowledge of tick-borne encephalitis virus (TBEV) circulation in the Netherlands, we conducted serosurveillance in roe deer (*Capreolus capreolus*) during 2017 and compared results with those obtained during 2010. Results corroborate a more widespread occurrence of the virus in 2017. Additional precautionary public health measures have been taken.

Tick-borne encephalitis virus (TBEV) was detected in the Netherlands during 2016. Retrospective screening of 297 roe deer (*Capreolus capreolus*) serum samples obtained during 2010 showed 6 samples contained TBEV-neutralizing antibodies. Five of these 6 serum samples had been obtained in Sallandse Heuvelrug National Park (Figure 1). A strain of the virus subtype from Europe (TBEV-EU) was subsequently detected in *Ixodes ricinus* ticks collected at this national park (*1*).

Shortly TBEV was reported to health professionals in the Netherlands, 2 probable autochthonous human tick-borne encephalitis (TBE) cases were identified, followed by a third case in 2017. The first case-patient probably got infected in the central part of the Netherlands in Utrechtse Heuvelrug National Park (Figure 1) (*2*); the second and third case-patients resided near Sallandse Heuvelrug National Park, where the TBEV-positive ticks were found (*3**,**4*). A tick removed by the first case-patient contained RNA of a virus strain more closely related to the TBEV-EU Neudörfl strain than to the TBEV-EU strain from Sallandse Heuvelrug National Park (*2*). This finding implied multiple infection sites and multiple strains of TBEV-EU in the Netherlands.

**Figure 1:**
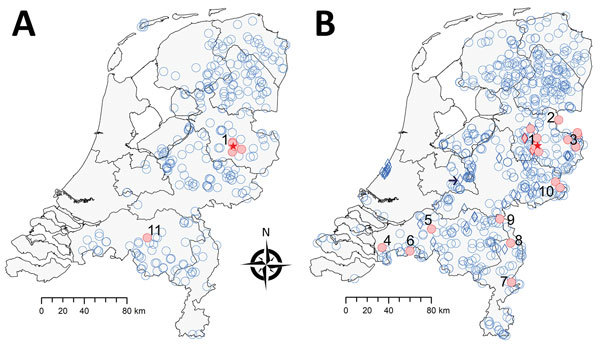
Geographic distribution of tick-borne encephalitis virus (TBEV) based on serosurveillance of roe deer, the Netherlands, during A) 2010 and B) 2017. Data for 2010 were reproduced from Jahfari et al. (*1*). Red indicates roe deer serum samples that showed positive results in the TBEV neutralization test, and blue indicates roe deer serum samples that showed negative results in this test or an ELISA. Numbers indicate confirmed or potential foci, and red stars indicate location of 2016 TBEV-RNA positive ticks in Sallandse Heuvelrug National Park. Circles indicate sites of random sampling, and diamonds indicate sites of purposive sampling. Arrow in the right map indicates location of Utrechtse Heuvelrug National Park. Maps were constructed by using Arc-GIS software (ESRI, https://www.esri.com).

Given these developments, there was a renewed need to locate potential TBEV foci and identify signs of increased TBEV circulation, in view of taking appropriate public health decisions. Therefore, we collected roe deer blood samples during 2017, tested them for TBEV and TBEV-neutralizing antibodies, and compared results with those from 2010 (*1*).

## The Study

Hunters submitted EDTA blood and serum samples by using the S-Monovette collection system (Sarstedt, https://www.sarstedt.com/en/home) from roe deer in locations across the Netherlands (Figure 1; Appendix 1). To enable comparison between years, most samples were requested through game management units by using the same method as in 2010, which had been designed for prevalence studies (random sampling). Samples from 592 roe deer were obtained. Furthermore, given the focal nature of TBE occurrence (*5*), specific game management units were asked during the year to supply extra samples (purposive sampling 48 samples), which were administered separately.

As during 2010 (*1*), we first screened 640 serum samples for TBEV-reactive antibodies by using a commercial ELISA (Immunozym FSME IgG all species with inactivated TBEV coating; PROGEN Biotechnik GmbH, https://www.progen.com). We then conducted a TBEV serum neutralization test (SNT) on ELISA-positive (>126 Vienna units/mL) or borderline (63–126 Vienna units/mL) samples. The serum dilution at which 50% of the Neudörfl strain (prototype TBEV-EU) is neutralized was calculated according to the method of Reed and Muench (*6*). At a 50% dilution >1:20, the SNT result was considered positive.

We also conducted PCR on RNA extracted from whole blood with primers as described (*7**,**8*) to detect early infection. A potential focus was defined by the presence of a TBEV SNT–confirmed or PCR-positive roe deer case. A confirmed focus was defined as a site with molecular evidence of the virus. Considering the home range size for roe deer, we arbitrarily considered a case within 10 km part of the same focus.

A total of 22/640 specimens had SNT-positive results: 17/592 (10/20 ELISA-positive and 7/55 ELISA-borderline cases) in the random sample and 5/48 (4/5 ELISA-positive and 1/8 ELISA-borderline cases) in the purposive sample (Table). No sample showed positive results by PCR. The SNT-confirmed cases occurred in the known focus of Sallandse Heuvelrug National Park (focus no. 1) and 9 potential foci (foci nos. 2–10) (Table; Figure 1). All 10 foci were detected by random sampling. Purposive sampling confirmed the known focus (no. 1).

**Table:** Detection of TBEV in roe deer, the Netherlands, 2017*

Province	No. (%) deer counted†	No. TBEV SNT–positive samples/no. tested		Identification no. of foci
		Random sampling	Purposive sampling	
Drenthe	10,728 (16)	0/99	NA	NA
Flevoland	2,358 (4)	0/37	NA	NA
Friesland	6,217 (10)	0/62	0/1	NA
Gelderland	10,687 (16)	2/97	0/12	10
Groningen	4,649 (7)	0/32	NA	NA
Limburg	4,674 (7)	3/46	0/1	7, 8, and 9
North Brabant	9,618 (15)	3/101	0/5	4, 5, and 6‡
North Holland§	935 (1)	0/0	NA	NI
Overijssel	9,933 (16)	9/87	5/18	1,¶ 2, and 3
South Holland	1,103 (2)	0/7	0/10	NA
Utrecht	2,485 (4)	0/16	0/1	NA
Zealand§	1,293 (2)	0/8	NA	NA
Total	64,680 (100)	17/592	5/48	NA

*NA, not applicable; SNT, serum neutralization test; TBEV, tick-borne encephalitis virus. †Provided by the game management units. ‡A fourth potential focus (no. 11) was identified by using serum samples obtained from roe deer during 2010 but not reconfirmed in 2017. §No hunting, only victims of traffic accidents. ¶Sallandse Heuvelrug National Park (the confirmed site, also identified by using serum samples obtained from roe deer during 2010).

We investigated probabilities of an increase in TBEV circulation and geographic expansion of TBEV by using random sampling results for 2010 and 2017 and R statistical software packages (https://www.r-project.org). Underlying assumptions were that SNT-confirmed roe deer cases had been infected with TBEV, and that there was no effect of hunter participation level. For roe deer, we used logistic regression with year, sex, age category (juvenile, immature, mature), and nutritional condition (good, moderate, poor) as independent variables to analyze whether year of sampling affected the likelihood of antibodies against TBEV (R scripts) (Appendix 2). We applied the Akaike information criterion for model reduction on all independent variables except year. The model was not improved by the other variables, which were then removed. We calculated the odds ratio for year and 95% profile (log) likelihood CI as 1.43 (95% CI of 0.59–4.01), which showed no clear effect of year. The results provide no evidence for an increase in roe deer cases in 2017 compared with 2010.

**Figure 2:**
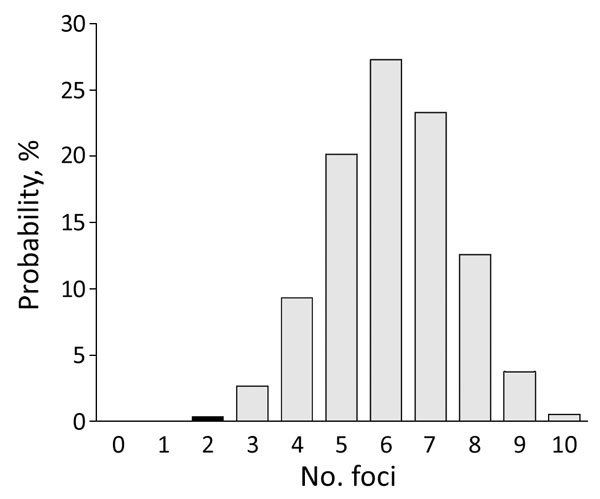
Probability distribution of number of potential foci containing tick-borne encephalitis virus expected to be detected during 2017 if only 297 of 590 roe deer samples had been submitted for testing, the Netherlands. Black column indicates the probability corresponding to the number of foci detected during the retrospective study of 297 samples obtained during 2010.

For focal diseases, such as TBE, sampling intensity might affect the detection rate for foci. Sampling intensity was greater in 2017 (592 events) than in 2010 (297 events). To investigate TBE expansion during 2010–2017, we randomly selected 297 events (samples) from the 592 events in the 2017 distribution (the study in 2017) and recorded the number of distinct foci that occurred. We repeated this procedure 100,000 times to obtain a probability distribution for the number of foci (Appendices 1, 2). The probability of obtaining <2 foci in 2017 was low (0.4%) (Figure 2). This finding indicates that the number of TBE foci probably increased during 2010–2017.

## Conclusions

Our study corroborates a more widespread occurrence of TBEV foci in the Netherlands than that identified on the basis of roe deer samples obtained during 2010. The lack of evidence for a major increase in roe deer exposure to TBEV could indicate these foci are recent, small, and possibly poorly established. Both focus size and sample distribution will affect detection and could explain why no foci were detected in Utrechtse Heuvelrug National Park or at site no. 11 during 2017. Identification of human cases and wildlife surveillance data are consistent with a general trend of geographic expansion of TBE that was concluded by an international working group on TBEV on the basis of data obtained from across Europe during 2007–2009 (*9*).

Many of the new potential foci we found are located near border areas with Germany or Belgium. Border districts in Germany are not reported to be risk areas for TBE. However, recent autochthonous human cases of TBEV infection were detected in Borken (2015) and Emsland (2016 and 2017) (*10*). In Flanders, wildlife had TBEV-neutralizing antibodies (*11**,**12*).

TBEV-infected areas are preferably identified through TBEV-specific antibodies in sentinel species (*13*). Roe deer have small home ranges, are often infested with *I. ricinus* ticks, seroconvert well (*14**,**15*), and are proven good sentinel species (*1**,**14**,**15*). However, if one considers serologic cross-reactivity among flaviviruses, potential foci identified need to be confirmed by other methods, such as PCRs for detecting TBEV in ticks from these sites (*1**,**16*).

These and other findings led to several public health measures in the Netherlands over the past 2 years. Microbiologists and clinicians are alerted regularly about TBE, its clinical appearance, and appropriate laboratory tests. Information on TBE is incorporated in tick bite prevention information, including on the website of the National Institute for Public Health and the Environment (https://www.rivm.nl/en). Professionals have been informed and reminded of measures to prevent tick bites. In the Sallandse Heuvelrug National Park area, inhabitants and general practitioners are specifically informed about TBE, including the availability of vaccines, by the Regional Public Health Services. We show that outcomes of surveillance in a sentinel wildlife species can directly contribute to public health interventions, which is an illustrative example of an effective One Health approach.

Appendix 1. Roe deer used in the study of tick-borne encephalitis virus, the Netherlands.

Appendix 2 Additional information on tick-borne encephalitis virus in roe deer, the Netherlands.
