# Supplementary material for: Tick-Borne Encephalitis Virus Antibodies in Roe Deer, the Netherlands
Source: Emerg Infect Dis. 2019 Feb;25(2):342–5. doi: 10.3201/eid2502.181386 (PMC6346459; doi:10.3201/eid2502.181386)
Supplement: Appendix 2 — Additional information on tick-borne encephalitis virus in roe deer, the Netherlands. [file 18-1386-Techapp-s2.pdf]

Article DOI: https://doi.org/10.3201/eid2502.181386

Tick-Borne Encephalitis Virus Antibodies in Roe Deer, the Netherlands

Appendix

Supplemental Material for R Script

```
library(readr)

setwd("…")

tbe <- read_csv("tbe.csv,"col_types = cols(

Datefound = col_date(format = "%d/%m/%Y"),

Datereceived = col_date(format = "%d/%m/%Y")

)

)

case <- ifelse(tbe$TBEResult = = "positive,"1,0)

noncase <- ifelse(tbe$TBEResult = = "negative," 1,0)

attach(tbe)

weight <- as.numeric(Weight)

AgeCategory1 <-factor (AgeCategory, levels = c("Juvenile," "Immature," "Mature"))

names(tbe)
```

#Effect of Year and Confounders on Cases: Binomial Logistic Regression Model

```
fit <-
glm(case~factor(Year)+factor(Gender)+AgeCategory1+factor(NutritionalCondition),family =
 binomial)

summary(fit)

cbind(exp(coef(fit)),exp(confint(fit)))

fit <-
```

```
glm(case~factor(Year)+factor(Gender)+AgeCategory1+factor(NutritionalCondition),family = binomial)
summary(fit)
cbind(exp(coef(fit)),exp(confint(fit)))
fit <-
glm(case~factor(Year)+factor(Gender)+AgeCategory1,family = binomial)
summary(fit)
cbind(exp(coef(fit)),exp(confint(fit)))
fit <-
glm(case~factor(Year)+factor(Gender)+factor(NutritionalCondition),family = binomial)
summary(fit)
cbind(exp(coef(fit)),exp(confint(fit)))
fit <-
glm(case~factor(Year)+AgeCategory1+factor(NutritionalCondition),family = binomial)
summary(fit)
cbind(exp(coef(fit)),exp(confint(fit)))
fit <-
glm(case~factor(Year)+AgeCategory1,family = binomial)
summary(fit)
cbind(exp(coef(fit)),exp(confint(fit)))
fit <-
glm(case~factor(Year)+factor(NutritionalCondition),family = binomial)
summary(fit)
cbind(exp(coef(fit)),exp(confint(fit)))
fit <-
```

```
glm(case~factor(Year)+factor(Gender),family = binomial)

summary(fit)

cbind(exp(coef(fit)),exp(confint(fit)))

fit <- glm(case~factor(Year),family = binomial)

summary(fit0)

cbind(exp(coef(fit)),exp(confint(fit)))
```

#Probability of Geographic Expansion: Probability Distribution (haard = focus)

```
tbe17 <- subset(tbe, Year =  = ”2017”)

detach(tbe)

names(tbe17)

n.haard <- NULL

for (i in 1:100000){

x <- sample(tbe17$Haard,297)

n.haard[i] <- length(names(table(x)))

y <- as.numeric(names(table(x)))[1]

if (y =  = 0) n.haard[i] <- n.haard[i]-1

}

table(n.haard)/100000

0:10%*%table(n.haard)/100000

barplot(table(n.haard)/100000)
```
